# Aerodynamic Effects of Time-Varying Corrugations on Dragonfly Wings in Flapping Flight

**DOI:** 10.3390/biomimetics9070433

**Published:** 2024-07-17

**Authors:** Dan Hou, Biao Tan, Binghao Shi, Zheng Zhong

**Affiliations:** 1 Department of Mechanical Engineering, Shanghai Maritime University, Shanghai 201306, China; 2 School of Science, Harbin Institute of Technology, Shenzhen 518055, China

**Keywords:** time-varying corrugations, flapping flight, aerodynamic performance

## Abstract

The aerodynamic effects of wing corrugation on insect flight have received widespread attention. However, there has hardly been any specific focus on dynamic changes to corrugation angle in the models. The flexible vein joints containing resilin in the wings of dragonflies and damselflies enable the longitudinal veins to rotate and thereby change the corrugation angles throughout flapping cycles. Therefore, a two-dimensional corrugated airfoil with time-varying corrugation angles is proposed and the aerodynamic performance is evaluated in terms of aerodynamic force, power and efficiency. The results indicate that the airfoil with time-varying corrugations outperforms the rigid one in terms of enhancing thrust and reducing power consumption. The aerodynamic performance of time-varying corrugated airfoils is optimal when the angle varies in a specific range, and an excessively large angle variation may have negative effects. In addition, excessive height or a negative leading edge of the corrugation can lead to a reduction in the thrust. A design concept for the 2D airfoil with time-varying corrugations is provided and the findings are of significance for enhancing the aerodynamic performance of biomimetic flexible flapping-wing vehicles.

## 1. Introduction

Corrugated morphology is a common structural feature of insect wings, especially dragonfly wings. As the longitudinal veins and cross-veins are not distributed in the same plane, concave or convex angles are formed at the junctions where they are connected. Thus, the cross section of the dragonfly wing shows a distinctive triangular wave pattern. The influence of corrugation on the wing’s structural performance is obvious, for it notably increases the moment of inertia of the cross section. Rees [1] introduced a beam model with triangular wave profiles and proposed that the corrugations could enhance the bending stiffness significantly by calculating the second moments of cross-section area. Subsequently, Sunada et al. [2] added that corrugation can enhance the warping moment of the wing section, which is the dominant resistance against the external moment for thin, corrugated dragonfly wings.

The specific impact of corrugation on flight aerodynamics is not well understood. Based on wind tunnel experiments, it is found that corrugated airfoils have superior aerodynamic performance compared to flat plates in gliding, particularly in terms of lift enhancement [3,4]. This remarkable result revealed that the surface morphological characteristics of insect wings are important factors in aerodynamics. With the development of CFD technology, the experimental results in Kesel [4] have been verified by Kim et al. [5] through numerical methods. More and more computational results indicate that the effects of corrugation are related to the Reynolds number (Re), as well as the geometric parameters of corrugation. It was found that the corrugated structure increased the lift coefficient at Re = 10,000, while it decreased the lift coefficient at Re = 5000 [6]. Studies on simplified corrugated camber airfoils revealed the importance of a corrugated camber in the high lift and low drag performance of dragonfly gliding flight [7]. Airfoils with specific corrugation amplitudes were found to exhibit superior aerodynamic performance compared to flat plates [8]. The gliding performances of a precise 3D corrugated dragonfly wing were studied and the V-shaped corrugation of the leading edge was found to be covered by separation vortices [9].

Compared to gliding, there are more unsteady aerodynamic effects in flapping flight. Considering hovering and forward flight, the aerodynamic performance of corrugated airfoils were studied and it was concluded that corrugations have a negative effect on lift and counteract the positive effects of camber deformation [10,11]. It was suggested that using a rigid flat plate to replace a real wing is a reasonable approximation. Leading-edge corrugation was found to be beneficial in enhancing the lift coefficient and reducing the drag coefficient when the flapping wings operate at a relatively higher angle of attack, while corrugation placed over the entire wing was not beneficial for aerodynamic efficiency [12]. The impacts of corrugations on aerodynamics during hovering flight have been revealed to be due to the asymmetric shape of the corrugations in down- and up- strokes [13]. The hovering aerodynamics of a corrugated wing were found to be significantly influenced by a combination of factors: the geometric characteristics, the distribution of corrugations, and the kinematic aspects of the wing’s motion [14]. In recent years, fluid–structure interaction methods have been applied to study the aerodynamic effects of flexible corrugated airfoils, and it is found that the flexibility of corrugations can improve lift or thrust to varying degrees [15,16].

In the above research, a multitude of factors such as the shape, distribution and camber deformation were considered in studying the corrugation effects. Over the past several decades, the development of experimental testing techniques has revealed much clearer images of the microscopic structure of insect wings. Notably, vein joints embedded with resilin are widely found in dragonfly and damselfly wings, endowing the corrugated angles with remarkable flexibility [17,18,19,20,21]. Resilin, a highly elastic biological protein, possesses a rather low elastic modulus and an ultimate strain that can reach 300% [22]. Mechanical tests and finite element analysis on individual joint as well as the entire wings showed that the more resilin present, the lower the torsional stiffness of the joint, and the larger the angle of rotation [19,23,24,25]. Thus, the corrugation angle can vary from time to time in response to the loads in flapping flight. And it is reasonable to believe that this change in angle will produce effects on the flight aerodynamics. As a result, a two-dimensional corrugated airfoil with time-varying angles is proposed and the specific aerodynamic impacts on flapping wings in flight are analyzed through the Computational Fluid Dynamics (CFD) method.

## 2. Materials and Methods

### 2.1. The Flapping Motion of the Airfoil

**Figure 1:**
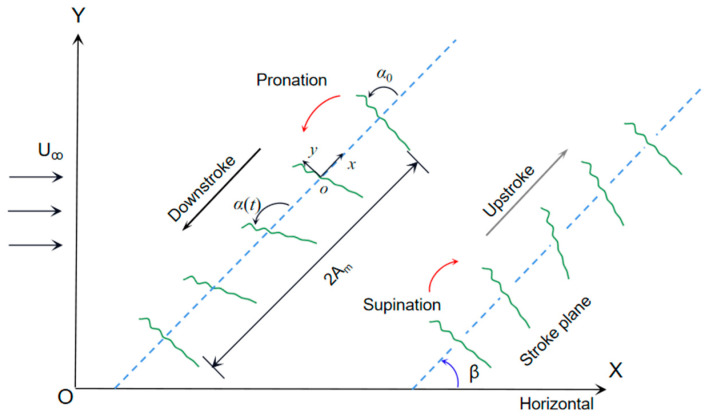
Sketches of the wing flapping motion. The stroke plane is inclined at *β* and the inflow velocity is U_ꝏ_. The wing rotates sharply to change the angle of attack before the end of each half-stroke, defined as pronation and supination.

The flapping motion of a dragonfly wing is shown in Figure 1. The wing flaps through an inclined stroke plane and the body is held almost horizontally [26,27]. The inertial coordinate system XOY is fixed on the ground and the body-fitted coordinate system *xoy* is fixed on the rotation center of the airfoil, which is located at 1/4 of the chord length *c*. The motions are approximated by the simple harmonic functions, as

(1) $$A(t)=A_{m}\cos(2\pi \;ft)$$

(2) $$\alpha (t)=\alpha_{0}-\alpha_{m}\sin(2\pi \;ft+\phi )$$

where A(t) is the translation displacement, $A_{m}$ is the amplitude, *α*(*t*) is the rotational angle of the chord relative to the stroke plane, $\alpha_{0}$ and $\alpha_{m}$ are the initial and amplitude of the rotational angle, ϕ is the phase difference between rotation and translation, and *f* is the flapping frequency.

### 2.2. Two-Dimensional Airfoil with Time-Varying Corrugations

Three different cross sections along the span of the dragonfly wing were extracted and their profiles were obtained through optical scanning [4]. The corrugated profiles at different locations exhibited the following characteristics: corrugations in the leading edge are obvious and tend to be flat in the trailing edge; the corrugation height from the root to tip of the wing decreases in a spanwise direction. Based on these observed shape characteristics, the zigzag airfoils are used widely to describe the corrugated airfoil in numerical studies.

**Figure 2:**
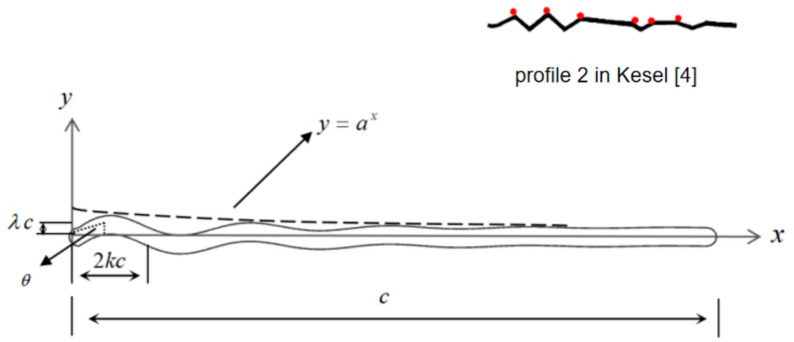
Schematic illustration of the 2D corrugated airfoil. By fitting the corrugation heights (red dots on the upper surface) distributed in the chord of profile 2 in Kesel [4], the value of a is determined to be 0.9.

Considering the continuous change in the corrugation angle caused by flexible joints, a sinusoidal corrugated airfoil is established as

(3) $$y(x)=\lambda c\cdot \sin\left(\frac{2\pi }{kc}x\right)\cdot a^{x},\begin{array}{cc} & 0\leq x\leq c \end{array}$$

where λ denotes the ratio of corrugation height to *c*, which decreases in *x* direction from the leading edge to the trailing edge by $a^{x}$ when a<1. When λ=0, the airfoil is a flat plate; *k* denotes the ratio of corrugation width to *c*. In this way, the angle of corrugation can be determined as tanθ=k/λ, as shown in Figure 2.

Based on the actual corrugated wing profiles, the following two conditions are ensured in parameter design. Firstly, there should be 6 to 8 corrugation angles from the leading edge to 0.7 c. Secondly, the height of the first corrugation angle should be 3–5% *c*. Thirdly, the height of each angle should decrease gradually from the leading edge to the trailing edge.

As y(*x*) is the initial shape of the airfoil, a function of time is required to define the change in the airfoil shape. The time-varying of the corrugation angle is not easy to describe, precisely due to the lack of experimental data. A simplification has been made in the variation of corrugation angles. The corrugation amplitude is defined to vary within a certain range according to the cosine law, corresponding to the flapping motion. Thus, the dynamic profile of the airfoil is defined as

**Figure 3:**
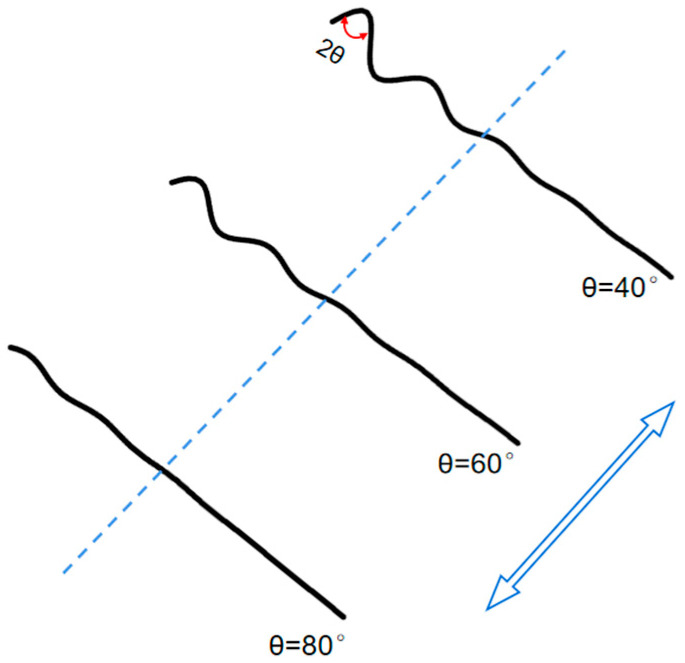
A diagram of the airfoil with varying corrugation angles in flapping motion.

(4) y(x,t)=y(x)[Acos(2πft+ψ)+B]

where ψ denotes the phase difference relative to the flapping motion in Equation (1). When ψ=0, λ is maximal at the beginning of the downstroke and minimal at the beginning of the upstroke. A and B are constants to modulate the translation of the cosine function, based on the given range of the angle variation in one flapping cycle. A diagram of the airfoil with time-varying corrugation angles in the flapping motion is shown in Figure 3.

### 2.3. The Flow Equations and Solution Method

The flow field is low-speed and the laminar fluid model is adopted, which has been proven to be capable of flapping wing calculations [28]. The governing equations of the flow are the incompressible unsteady Navier–Stokes equations, which are defined as

(5) ∇·u=0∂u∂t+u·∇u=−∇p+1Re∇2u

where **u** is the non-dimensional fluid velocity field, *p* and *t* are the non-dimensional fluid pressure and time, respectively, and Re is the Reynolds number. The velocity–pressure coupling equations are solved by an algorithm with second-order accuracy in both space and time. The second-order upwind scheme is used for the discretization of convective fluxes in the momentum equation. The pressure term is discretized with the second-order scheme, and the second-order implicit scheme is used to solve the transient term.

**Figure 4:**
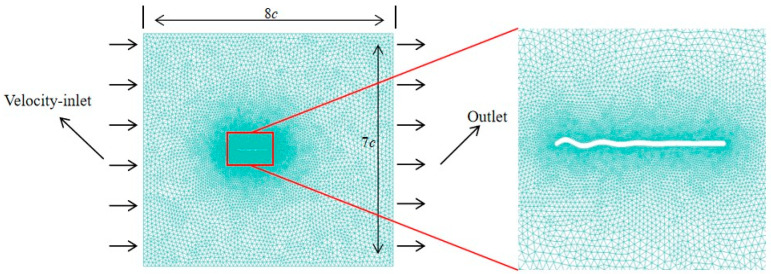
The computational domain with grid and boundary conditions.

For far-field boundary conditions, the velocity components are specified as free-stream conditions at the inflow boundary and pressure is set to the free-stream static pressure at the outflow boundary. On the wing surface, impermeable wall and non-slip conditions are applied and the pressure is obtained through the normal component of the momentum equation written in the moving grid system. The calculation domain and boundary conditions are shown in Figure 4. The mesh is finest surrounding the wing in the high-density region, and the mesh grows in size towards the far field. The fluid velocity components and pressure at discretized grid points for each time step are available when the Navier–Stokes Equation (5) are numerically solved. The aerodynamic forces acting on the wing are calculated from the pressure and the viscous stress exerted by the fluid on the wing surface.

### 2.4. Parameters in Calculation

The aerodynamic performance is characterized by the lift coefficient $C_{L}$ and thrust coefficient $C_{T}$ as

(6) $$C_{L}=F_{L}/0.5\rho U^{2}c$$

(7) $$C_{T}=F_{T}/0.5\rho U^{2}c$$

where ρ is the fluid density, U is the referenced velocity, $F_{L}$ is the lift, and $F_{T}$ is the thrust, which is numerically equal to the negative of the drag; the directions of lift and thrust are defined as vertical and horizontal (Y and −X in Figure 1) in the simulation. By taking the average of the aerodynamic force within a stable flapping cycle, the time-averaged lift coefficient and thrust coefficient are obtained as

(8) $$C_{Lm}=\frac{\frac{1}{T}\int_{t}^{t+T}F_{L}(t)dt}{0.5\rho U^{2}c}$$

(9) $$C_{Tm}=\frac{\frac{1}{T}\int_{t}^{t+T}F_{T}(t)dt}{0.5\rho U^{2}c}$$

The consumed power P(t) includes power in translation (Equation (1)) and power in rotation, Equation (2), which is defined as

(10) $$P(t)=-\left[F_{L}(t)\cdot {\dot{U}}_{Y}(t)-F_{T}(t)\cdot {\dot{U}}_{X}(t)+M(t)\cdot \dot{\alpha }(t)\right]$$

where ${\dot{U}}_{x}(t)$ and ${\dot{U}}_{Y}(t)$ are the translation velocities of the fluid in the thrust and lift directions, respectively, and M(t) is the instantaneous generated torque about the rotational axis. As in the motion shown in Figure 1, they can be calculated as $U_{X}=\dot{A}(t)\cos\beta +U$ and $U_{Y}=\dot{A}(t)\sin\beta$. The power coefficients $C_{P}$ and $C_{\mathrm{Pm}}$ are

(11) $$C_{P}=P/0.5\rho U^{3}c$$

(12) $$C_{Pm}=\frac{\frac{1}{T}\int_{0}^{T}P(t)dt}{0.5\rho cU^{3}}$$

In flapping flight, the lift efficiency $\eta_{L}$ and thrust efficiency $\eta_{T}$ can be used to describe the aerodynamic performances [29]. They are defined using the power required for lift and thrust divided by the aerodynamic power required for moving the wing in the air. From the equations above, the efficiencies are obtained as

(13) $$\eta_{L}=\frac{-\frac{1}{T}\int_{t}^{t+T}C_{L}(t)\cdot U_{Y}(t)dt}{C_{\mathrm{Pm}}U}$$

(14) $$\eta_{T}=\frac{\frac{1}{T}\int_{t}^{t+T}C_{T}(t)\cdot U_{X}(t)dt}{C_{\mathrm{Pm}}U}$$

Based on the previous studies [26,27,30], these parameters are used in the following analysis: *f* = 33 Hz, β=3π/8, $A_{m}=1.5c$, $\alpha_{0}=\pi /2,$ $\alpha_{m}=-\pi /4$ and ϕ=0. In addition, the chord length is c = 10 mm, the thickness is 0.03 c, and kinematic viscosity of the air ʋ = 1.516 × 10^−5^ m^2^/s. The forward flight speed U and chord length *c* are taken as the reference velocity and reference length, U = 2.3 m/s and Re = Uc/ʋ ≈ 1500.

## 3. Results and Discussion

### 3.1. Grid Sensitivity Test, Time-Step Convergence and Code Validation

**Figure 5:**
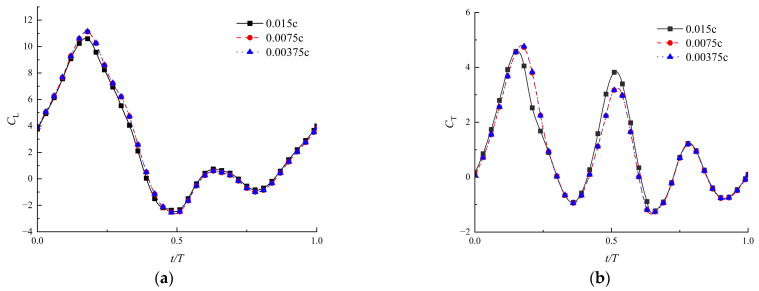
Coefficients of lift (**a**) and thrust (**b**) for three test grids (Re = 1500, λ=0.04, $\theta \in [40^{\circ },\;50^{\circ }]$).

The Fluent software version 8.5 was applied to solve Equation (5) and to simulate the flow field based on the control-volume method. The flapping motion and deformation were achieved through dynamic mesh technology. Firstly, to ensure computational accuracy and to enhance the computational efficiency, a grid independence verification was conducted. Three different grid sizes were selected for testing: the minimum mesh sizes around the wing, which were 0.015 *c*, 0.0075 *c*, and 0.00375 *c*, respectively, refined uniformly by a factor of 2. The maximum size of the cell was ten times larger. As a result, the total grid numbers were 10,000, 25,000, and 50,000, respectively. The lift coefficient of the flapping wing with time-varying corrugations described by Equation (4) was calculated, and the results are shown in Figure 5. It can be observed that the time-variation of the lift coefficient over a flapping cycle almost overlapped when the boundary layer cell sizes were 0.0075 *c* and 0.00375 *c*, respectively. Therefore, considering the requirements for accuracy and computational efficiency, the calculations in this paper are conducted using a grid with a minimum cell size of 0.0075 *c*.

**Figure 6:**
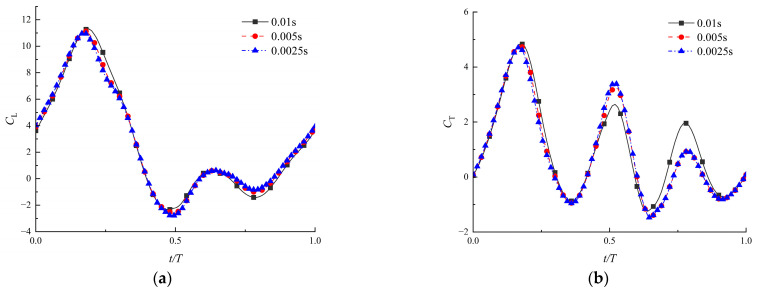
Coefficients of lift (**a**) and thrust (**b**) for three time-steps (Re = 1500, λ=0.04, $\theta \in [40^{\circ },\;50^{\circ }]$).

Secondly, it is also necessary to determine the independence of the time step with the results for unsteady aerodynamic analysis. As the mesh needs to move and reconstruct in each time step, the size of the time step can be preliminarily determined according to the minimum mesh size and moving speed. In this range, three time-steps were selected for comparison to demonstrate that the time step will not influence the accuracy, as shown in Figure 6. The obtained aerodynamic coefficients all reached a stable state after four cycles. When the time steps were 0.005 s and 0.0025 s, the curves were close enough to be a coincidence. Therefore, the time step of 0.005 s was used in the calculation, which could ensure the accuracy of the results.

**Figure 7:**
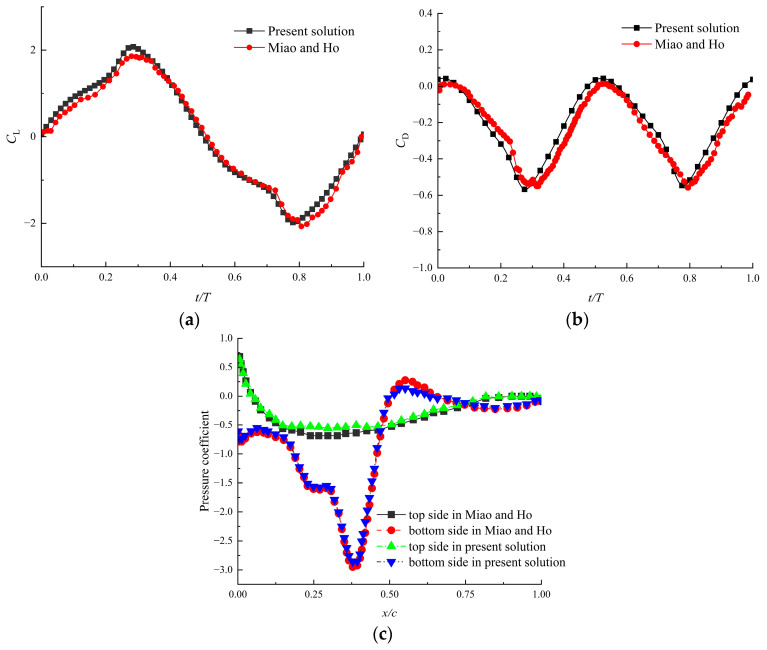
The comparison with previous numerical results ($a_{0}=0.3$, k=2, and $h_{0}=0.4$ in Miao and Ho [31]): (**a**) lift coefficient, (**b**) drag coefficient and (**c**) pressure coefficient.

Thirdly, to verify the correctness and effectiveness of the numerical method in this paper, the flapping wing model with given flexure proposed by Miao and Ho [31] was calculated. The airfoil used was NACA0014, which underwent a plunge motion with Re=10,000. The aerodynamic coefficients of the flapping airfoil were obtained and compared with the previous results, as shown in Figure 7. It was indicated that the force coefficients matched well with the previous studies and that they varied over time in a similar pattern. Therefore, the method adopted in this paper can be used to solve the aerodynamic forces of flexible flapping airfoils. The errors presented may be due to the differences in the setting of parameters such as mesh size, time step, etc. In addition, the different solvers in the calculation also affected the results.

### 3.2. Effects of Time-Varying Corrugations at Different Phase Angles

**Figure 8:**
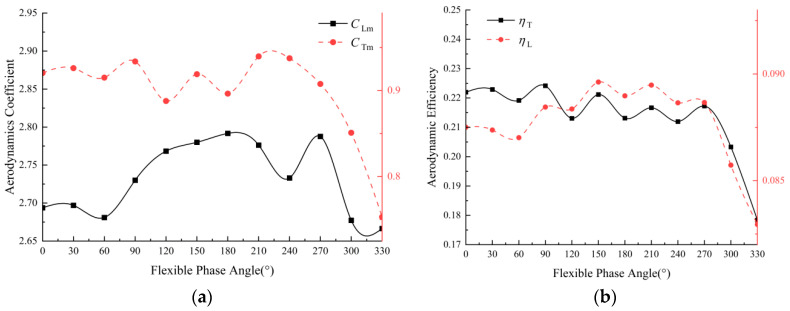
Aerodynamic force coefficients (**a**) and efficiencies (**b**) under different phase angles (Re = 1500, λ=0.04, $\theta \in [40^{\circ },\;50^{\circ }]$).

As shown in Figure 8, the $C_{\mathrm{Lm}}$ varies within a relatively small range, from 2.65 to 2.8, as does the phase angle ψ within the range of 0–360°, while $C_{\mathrm{Tm}}$ decreases obviously when $\psi >270^{\circ }$. Generally, the phase angle has a more significant effect on thrust and thrust efficiency. To comprehensively understand the aerodynamic performance of flapping wings, the lift efficiency and thrust efficiency are shown in Figure 8b. It demonstrates that the efficiencies perform well when the angle is 150°. Consequently, considering both forces and efficiencies, the phase angle is fixed to be 150° for all the calculations in the following study.

### 3.3. Effects of Time-Varying Corrugations at Different Reynolds Numbers

**Figure 9:**
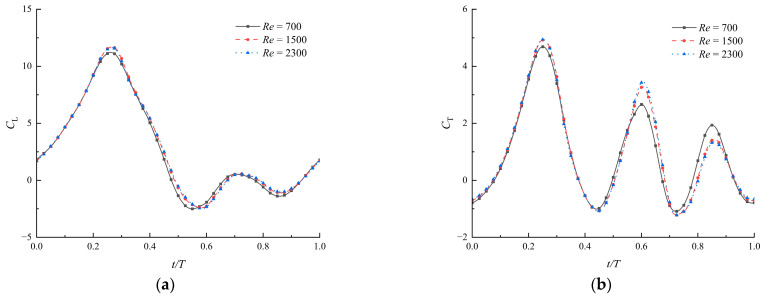
Coefficients of lift (**a**) and thrust (**b**) under different Reynolds numbers ($\psi =150^{\circ }$, λ=0.04, $\theta \in [40^{\circ },\;50^{\circ }]$).

The actual velocity of the air is the composition of velocities in flapping and forward flight. As introduced in Section 2.4, the velocity is the forward direction, ranging from *U* − *U*_max_ to *U* + *U*_max_, where *U*_max_ is the maximal flapping velocity. With the defined parameters, the velocity is obtained between 1.1 m/s and 3.2 m/s, and the Reynolds numbers are approximately 700 and 2300. Within this specified range, Re = 700 and Re = 2300 are added in the comparison, analyzing the impacts of Reynolds numbers on the aerodynamic performances of the flapping airfoils. The coefficients of lift and thrust are shown in Figure 9. It can be observed that when Re = 1500 and Re = 2300, there is minimal variation between the corresponding curves. The slight difference in lift could be due to the effective angles of attack being small at Re = 700.

Both the power consumption coefficients and efficiencies show minor variations in Table 1. The lift efficiency is basically unchanged at the three Reynolds numbers. In general, the change in aerodynamic force is not significant, as the Reynolds number varies within this range. This indicates the reasonability of using a two-dimensional airfoil to assess the three-dimensional flapping flow field in this paper. It is noticed that in the simulations, the thrust efficiencies reached over 20%, while the lift efficiencies were much lower. Certainly, it is necessary to further study the performances under different forward flight speeds.

### 3.4. Effects of Time-Varying Corrugations with Different Angles

Through meticulous angle measurements of the chordwise profiles demonstrated in experiments and the zigzag airfoils employed in numerical calculations, the size of the corrugation angles was found to range from 80° to 160°. Corresponding to the airfoil shown in Figure 1, the corrugation angle $\theta \in [40^{\circ }\text{–}80^{\circ }]$ is specified in the paper.

**Figure 10:**
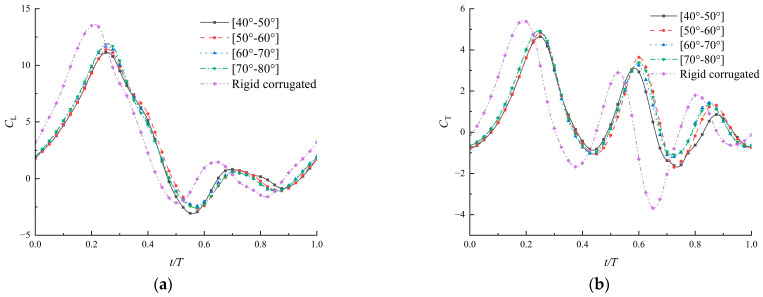
Coefficients of lift (**a**) and thrust (**b**) of corrugated airfoils with angles varying within a range of 10° ($\psi =150^{\circ }$, λ=0.04, Re=1500).

Firstly, the variation range of the corrugation angle is set to be 10^°^ during a flapping cycle. Four different wings with corrugation angles in the ranges of [40°–50°], [50°–60°], [60°–70°], and [70°–80°], respectively, are applied to compare the flapping aerodynamic performances. A set of rigid wings is also included, the corrugation angles of which do not change during the flapping cycle (the first corrugation angle is 40°). The time variations of the lift coefficient and the thrust coefficient for these airfoils are shown in Figure 10. In addition, the time-averaged power coefficient and force efficiencies are listed in Table 2.

The airfoils with time-varying corrugations show a similar high peak of aerodynamic forces immediately after the wings turn to decelerate. All four groups of airfoils with time-varying corrugations achieved their maximum lift and thrust at T/4, and the airfoil of [70°–80°] exhibited the highest force coefficients at that moment. The average coefficients increase gradually as the corrugation angle increases, especially the thrust coefficient. Compared with the first airfoil of [40°–50°], the average thrust of the other three airfoils is increased by 18%, 23%, and 30%, respectively. Therefore, within this variation range of 10°, larger angles can optimize the aerodynamic forces. Taking power consumption into account, an increase in the corrugation angle results in a thrust efficiency increase of 28%.

**Figure 11:**
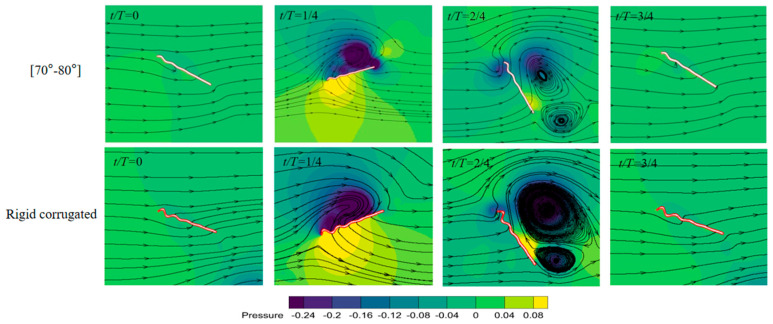
Pressure contours of the airfoil with time-varying angle in [70°–80°] and rigid corrugated airfoil in a flapping cycle.

The rigid airfoil reaches maximum aerodynamic forces earlier than airfoils with time-varying corrugations in Figure 10. This may be related to the adhesion time of the leading edge vortex on the airfoil. The rigid airfoil produces the largest lift and the lowest thrust on average. In particular, the thrust is about 11–32% lower than that of airfoils with time-varying corrugation. As shown in Figure 11, there is a remarkable pressure difference between upper and lower surfaces in downstroke, contributing to the lift. The thrust in upstroke is almost negative, and should be related to the large area of high pressure in front of the airfoil in upstroke. In addition, the power consumption of the rigid airfoil is highest, 20–28% higher than that of airfoils with time-varying corrugation. This directly leads to a decrease in aerodynamic efficiencies, especially with a maximum 30% reduction in thrust efficiency. It is concluded that the time-varying corrugation angles allow the airfoil to more effectively manipulate and utilize the airflow for forward momentum in upstroke.

**Figure 12:**
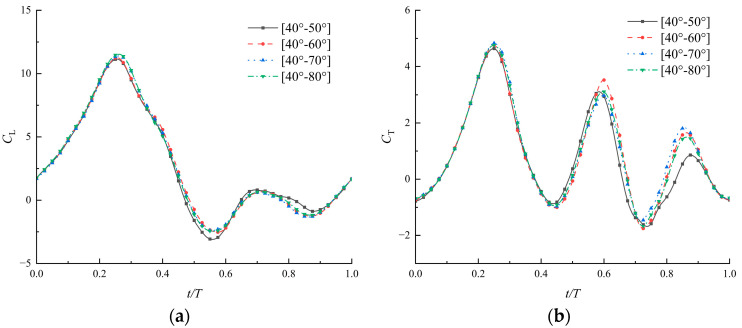
Coefficients of lift (**a**) and thrust (**b**) of airfoils with time-varying corrugated angles in different ranges ($\psi =150^{\circ }$, λ=0.04, Re=1500).

To further investigate the impacts of angle variation range within a flapping cycle, the airfoils are divided into groups with the corrugation angle varying in the ranges of [40°–60°], [40°–70°], and [40°–80°]. The corresponding angle variation ranges are 20°, 30°, and 40° individually. Figure 12 presents the results of the aerodynamic performances of the above airfoils with time-varying corrugations, and the time-averaged coefficients are listed in Table 3. The results indicate that the lift coefficient and thrust coefficient over time are very similar across the three groups, showing better aerodynamic forces compared with [40°–50°]. However, the aerodynamic force does not increase without limit as the angle increases. Too-large variations such as [40°–80°] in corrugation angles during the flapping cycle do not necessarily yield better aerodynamics. The rapid change in angle shape would affect the stability of airflow on the surface. The results could also explain why there are spikes growing on the flexible joints of the soft damselfly wings, which serve to prevent excessive rotation during flapping [19]. To improve both lift efficiency and thrust efficiency, other deformations, such as the bending of the section, may also need to be considered.

### 3.5. Effects of Time-Varying Corrugations under Different Heights

**Figure 13:**
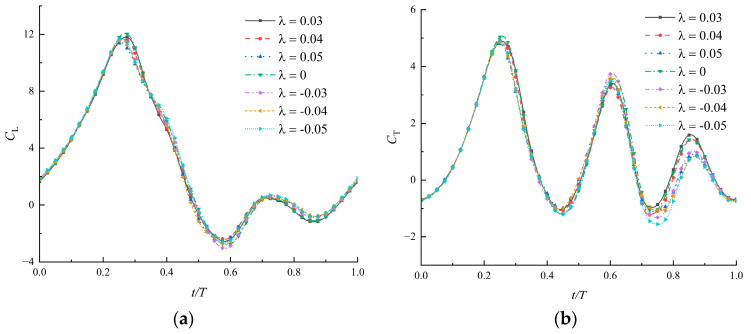
Coefficients of lift (**a**) and thrust (**b**) of airfoils with time-varying corrugations under different heights ($\psi =150^{\circ }$, $\theta \in [60^{\circ },\;70^{\circ }]$, Re=1500).

In this section, the influence of the size and direction of the initial corrugation height λ on the results is studied. The aerodynamic coefficients of seven airfoils with λ ranging from −0.05 to 0.05 are shown in Figure 13 and the time-average values are listed in Table 4. The first corrugation is convex upwards when λ>0 and concave downwards when λ<0 in Equation (3). The corresponding airfoils are called the positive airfoil and negative airfoil. There is a flat plate when λ=0.

As the direction and height of the corrugation change, the force curves are very similar and the main difference is in the thrust during the last quarter of the cycle. This results in a sequence from high to low in the average thrust in positive airfoils, flat airfoils, and negative airfoils. However, when the corrugation of the positive airfoil is too high (λ>0.03), the thrust begins to decrease. The power consumption of the flat airfoil is highest. It again shows that the flexibility of corrugation can play a leading role in reducing power consumption. Thrust efficiency is more sensitive to corrugation height than lift efficiency. For both positive and negative airfoils, the thrust efficiency varies within a 20% range as the corrugation height increases from 0 to 0.05. The corrugation height determines the thickness of the airfoil in the leading edge. The thinner airfoil helps improve thrust, which is consistent with Ashraf et al. [32], studying the thrust performance of NACA airfoils in flapping motion.

**Figure 14:**
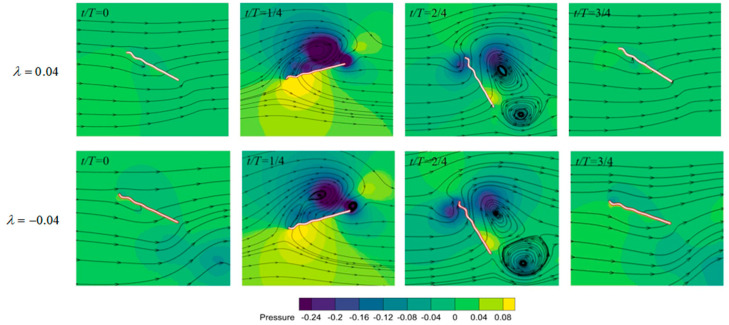
Pressure contours of the positive airfoil (λ=0.04) and negative airfoil (λ=−0.04) in a flapping cycle.

The pressure contours of the positive airfoil and negative airfoil are shown in Figure 14. For the airfoil with a positive leading-edge corrugation, there is an obvious low-pressure region attached to the upper surface at the location of maximum velocity during the downstroke. This is likely the reason why its lift performance is slightly superior to that of the negative airfoil. The difference in thrust curves shown in Figure 13b should come from the large area of high pressure in front of the negative airfoil during the last quarter cycle. Therefore, airfoils with different corrugation shapes in up- and downstrokes may help in improving the thrust efficiency.

## 4. Conclusions

Both corrugation and flexible vein joints are key structural features commonly found in insect wings. In this study, a 2D corrugated wing with time-varying corrugations is explored with consideration of their synergistic effects. By conducting a comprehensive analysis of the aerodynamic properties during forward flapping flight, the following conclusions are obtained.

Taking into account the overall aerodynamic performance, time-varying corrugated airfoils outperform the rigid ones in enhancing thrust and thrust efficiency. The aerodynamic performance of airfoils with time-varying corrugations is optimal when the angle varies in a specific range, such as 60° to 80°. However, an excessively large angle variation (e.g., 40° to 80°) during one cycle may have negative effects. Excessive height or negative corrugation can lead to reduced thrust. The sequence from high to low in thrust goes from positive airfoils to flat airfoil to negative airfoils. However, when the corrugation of the positive airfoil is too high (λ>0.03), the thrust begins to decrease.

Flexible corrugated airfoils hold potential for increasing the thrust and reducing energy consumption in flapping flight, critical for improving the propulsive efficiency of biomimetic flapping-wing vehicles. For future research, a more comprehensive fluid–structure interaction analysis will be essential, considering both the dynamic changes in corrugation angle and the bending deformation of the wing.

## Figures and Tables

**Table 1:** The time-averaged coefficients and efficiency of aerodynamic forces of the airfoil under different Reynolds numbers ($\psi =150^{\circ }$, λ=0.04, $\theta \in [40^{\circ },\;50^{\circ }]$).

Re	700	1500	2300
$C_{Lm}$	2.663	2.823	2.810
$C_{Tm}$	0.946	0.961	0.970
$C_{Pm}$	4.212	4.276	4.221
$\eta_{L}$	0.082	0.082	0.082
$\eta_{T}$	0.236	0.240	0.242

**Table 2:** The time-averaged coefficients and efficiencies of aerodynamic forces of airfoils with angles varying within a range of 10° ($\psi =150^{\circ }$, λ=0.04, Re=1500).

	[40°–50°]	[50°–60°]	[60°–70°]	[70°–80°]	Rigid Corrugated
$C_{Lm}$	2.698	2.779	2.823	2.801	3.387
$C_{Tm}$	0.780	0.918	0.961	1.016	0.693
$C_{Pm}$	4.008	4.153	4.277	4.280	5.122
$\eta_{L}$	0.080	0.081	0.085	0.087	0.100
$\eta_{T}$	0.194	0.229	0.239	0.248	0.173

**Table 3:** The time-averaged coefficients and efficiencies of aerodynamic forces of airfoils with angles varying within different ranges ($\psi =150^{\circ }$, λ=0.04, Re=1500).

	[40°–50°]	[40°–60°]	[40°–70°]	[40°–80°]
$C_{Lm}$	2.698	2.730	2.774	2.734
$C_{Tm}$	0.780	0.962	0.924	0.926
$C_{Pm}$	4.008	4.137	4.213	4.188
$\eta_{L}$	0.080	0.080	0.081	0.082
$\eta_{T}$	0.194	0.230	0.240	0.230

**Table 4:** The time-averaged coefficients and efficiencies of aerodynamic forces of airfoils with time-varying corrugations under different heights ($\psi =150^{\circ }$, $\theta \in [60^{\circ },\;70^{\circ }]$, Re=1500).

λ	−0.05	−0.04	−0.03	0	0.03	0.04	0.05
$C_{Lm}$	2.958	2.790	2.827	2.888	2.756	2.823	2.790
$C_{Tm}$	0.808	0.901	0.912	0.971	1.046	0.961	0.905
$C_{Pm}$	4.276	4.114	4.260	4.419	4.272	4.276	4.088
$\eta_{L}$	0.082	0.083	0.084	0.085	0.083	0.082	0.082
$\eta_{T}$	0.202	0.225	0.227	0.257	0.261	0.240	0.225

## Data Availability

The original contributions presented in the study are included in the article, further inquiries can be directed to the corresponding author.
